# Sensitive and rapid detection of tetrodotoxin based on gold nanoflower-and latex microsphere-labeled monoclonal antibodies

**DOI:** 10.3389/fbioe.2023.1196043

**Published:** 2023-05-16

**Authors:** Yongming Huang, Aidi Xu, Yang Xu, Huijuan Wu, Menghan Sun, Lakshani Madushika, Rongzhi Wang, Jun Yuan, Shihua Wang, Sumei Ling

**Affiliations:** The Ministry of Education Key Laboratory of Biopesticide and Chemical Biology, Fujian Key Laboratory of Pathogenic Fungi and Mycotoxins, School of Life Sciences, Fujian Agriculture and Forestry University, Fuzhou, China

**Keywords:** tetrodotoxin, monoclonal antibody, immunochromatography strip, detection, latex microsphere, gold nanoflower

## Abstract

Tetrodotoxin (TTX) could result in serious diseases due to its extremely high neurotoxicity. Thus, it is of great importance to measure TTX for food safety. In this study, an anti-TTX monoclonal antibody with good specificity and high affinity was used to develop the immunochromatographic test strips (ICTS). Gold nanoflower (AuNF) with multiple branches and latex microsphere (LM) with large particle size as signal reporters were employed for improving the sensitivity of test strips. Both AuNF and LM probes are stable, and the developed ICTS were specific to TTX, demonstrating no cross-reactivity with other marine toxins. The linear range of AuNF- and LM-based strips for TTX was 9.49–330.98 ng/mL and 5.40–443.19 ng/mL, respectively. The limit of detection (LOD) of AuNF- and LM-based strips was determined to be 9.49 ng/mL and 5.40 ng/mL, respectively. In summary, the developed ICTS based on AuNF and LM signal probes displayed enhancement of sensitivity and provided rapid and specific detection of TTX.

## 1 Introduction

Tetrodotoxin (TTX) derived from pufferfish is considered a non-protein marine toxin (Mebs et al., 1995). TTX causes paralysis by selectively coupling to voltage-gated sodium ion channels which interfere with nerve transmission (Bane et al., 2014). As a highly toxic neurotoxin, it causes serious diseases such as headache, muscle weakness, numbness in the mouth and vomiting, and even fatal respiratory or heart failure (Denac et al., 2000; Noguchi and Ebesu et al., 2001; Hwang and Noguchi et al., 2007). Previous studies have revealed that only 0.5–3 mg of TTX can lead to death in adults, and the LD_50_ of mice injected intraperitoneally is 80 μg/kg (Chain et al., 2017). Accidental consumption of pufferfish causes many cases of poisoning every year (Shkembi et al., 2021). In 2008, a total of 141 patients were hospitalized, with a mortality rate of up to 12% in Bangladesh (Islam et al., 2011). Hence, it is urgent to develop specific, sensitive, and fast assays for TTX detection.

Currently, the most commonly used methods for TTX detection include high-performance liquid chromatography (HPLC), surface plasmon resonance (SPR), liquid chromatography-tandem mass spectrometry (LC-MS/MS), fluorometric assay, enzyme-linked immunosorbent assay (ELISA), gas chromatography-mass spectrometry (GC-MS), mouse bioassay, and thin-layer chromatography (TLC) (Asakawa et al., 2000; Huang et al., 2008; Bane et al., 2016; Hayashida et al., 2003; Kao, 1966; Ling et al., 2015; Nunez et al., 1976; O'Leary et al., 2004; Vaisocherova et al., 2011). However, these experimental methods either require expensive equipment or highly trained personnel and significant laboratory infrastructure. The Lateral flow immunochromatography strip (LFICS), a popular and promising point-of-care testing (POCT) strategy with the outstanding features of low cost, high sensitivity, simplicity, speediness, and high specificity, has been used for the rapid determination of residues and contaminants in foods and environments (Liang et al., 2022). However, traditional competitive LFICS usually uses gold nanoparticles (AuNPs) as signal reporters to detect small molecules (Ling et al., 2020). The sensitivity of AuNPs is relatively low due to their insufficient brightness and small particle size (Shen et al., 2017). Recently, researchers have been working on building different enhanced signal reporters to increase the sensitivity of LFICS and the precision of medical treatments (Loynachan et al., 2018; Li et al., 2021a; Li et al., 2023; Lu et al., 2023). For example, WS_2_, MoS_2_, gold nanoflower (AuNF), and latex microsphere (LM) have already been used, and these materials have the advantages of high surface-to-volume ratio and easy preparation (Chen et al., 2023; Zhang et al., 2023). WS_2_ and MoS_2_ are often used in conjunction with fluorescence reporters, while AuNF and LM can be used as reporters themselves (Yang et al., 2015; Zuo et al., 2016). In addition, it has been shown that the colloidal and optical properties are very important to the sensitivity of the test strip (Ji et al., 2015). AuNF with the advantages of colloid stability and high optical extinction was introduced to increase the sensitivity of test strips (Petrakova et al., 2019). Moreover, AuNF with a larger specific surface area could improve the immobilization yield of antibodies. Previous findings showed that the sensitivity of LFICS based on AuNF for the testing of lead (Pb) ions was improved (Jia et al., 2022). Ji et al. reported that AuNF with advanced optical and colloidal properties exhibited more sensitivity by 10 times than AuNP for AFB_1_ detection. Latex microsphere with the advantages of good optical brightness and stability has gained great attention. As a sensitive label, LMs were applied in many areas. For example, a fluorescent retrograde tracer prepared by labeling LMs with rhodamine was used in the peripheral nervous system (Colin et al., 1989). An immunoassay strip using LMs as an enhanced signal reporter was sensitive (LOD = 25 ng/mL), reliable, and rapid for SARS-CoV-2 antigen testing (Shen et al., 2017). Xu et al. and Ling et al. used LM- and AuNP-based strips to detect cadmium ions, respectively, showing that the sensitivity of the LM-based strip is approximately seven times that of the AuNP-based strip (Ling et al., 2021; Xu et al., 2021).

In this study, we prepared flower-like gold nanoflower and latex microsphere probes to label monoclonal antibodies for developing immunochromatographic test strips for on-site and rapid TTX detection. To the best of our knowledge, this is the first study to develop the LM-based strips with LM as enhanced signal reporters for the detection of TTX in food.

## 2 Materials and methods

### 2.1 Materials

Hydroquinone (C_6_H_6_O_2_), chloroauric acid (AuCl_4_H), trisodium citrate dihydrate (C_6_H_5_Na_3_O_7_), and PEG 20000 were bought from Macklin (Shanghai, China). The NC membrane, sample pad, conjugated pad, polyvinylchloride (PVC) backing card, and absorbance pad were purchased from Jieyi Biotechnology Co., Ltd. (Shanghai, China). Latex microspheres were purchased from Bans Laboratories, Inc. (Indiana, United States). TTX (≥95% pure) was purchased from CATO Research Chemicals Inc. (Guangzhou, China), and anti-TTX-monoclonal antibodies (anti-TTX mAb) from hybridoma cell 5B9 were produced by us and stored in our lab (Ling et al., 2015). Anti-TTX mAb had no cross-reactivity against saxitoxin acetate (STX), conotoxin (CTX), brevetoxin (BTX), pectenotoxin (PTX), and microcystin (MC) (Ling et al., 2015). μ-Conotoxin KIIIA (μ-CTX KIIIA), αB-conotoxin VxXXIVA (αB-VxXXIVA-CTX), APETx_2 (κ-actitoxin-Ael2a, UniProt P61541), BDS-I (∆κ-actitoxin-Avd4a, UniProt P11494), and sea snake neurotoxins-311 (SN311) (≥85% pure) were prepared by Sangon Biotech (Shanghai) Co., Ltd. OA (≥98% pure) was purchased from Sigma-Aldrich (St, Louis, MO, United States).

### 2.2 Preparation of AuNF probes

The AuNF solution was prepared as described previously (Zhang et al., 2019). First, in order to synthesize AuNFs, AuNPs were used as seeds. Briefly, 100 mL of ddH_2_O, appropriate amounts of 1 M NaOH, 750 μL chloroauric acid, 500 μL AuNP solution, 300 μL 1% trisodium citrate dihydrate solution, and 1 mL of 0.03 M hydroquinone solution were added under stirring conditions. The AuNF probes were synthesized as described previously (Ling et al., 2021). AuNF solution (10 mL), anti-TTX mAb, and K_2_CO_3_ in optimal amounts were added under low-speed stirring. After stirring for 1 h, 1% BSA was added to the solution, and PEG 20000 (0.5%) was added after stirring for 45 min. Finally, the solution was stirred continuously for 45 min and placed at 4°C overnight. To obtain the AuNF probe, the aforementioned solution was centrifuged at 13,000 r/min followed by resuspension of precipitation and further storage at 4°C.

### 2.3 Preparation of LM probes

Based on the previously published paper, a latex microsphere (LM) probe was prepared (Ling et al., 2022). In a nutshell, 15 μL LMs were dissolved in MES activation buffer (1 mL, pH 6.0) and then centrifuged for 10 min at 4°C at 13,000 r/min. Then, the carboxyl groups on the LM surface were activated by 1.5 mg EDC and incubated at 25°C followed by centrifugation at 180 r/min for 15 min. After the LM solution was activated, it was centrifuged at 13,000 r/min. A measure of 0.5 mL of glycine buffer (pH 6.5) was added to resuspend the precipitate and then centrifuged at 13,000 r/min. Anti-TTX mAb (15 μL) were added to the LM solution after 0.5 mL of glycine buffer (pH 6.5) was added to the precipitate. After coupling the reaction for 4 h at 25°C, the solution was centrifuged at 12,000 r/min for 10 min and then blocked by adding 0.5 mL glycine buffer (pH 6.5). Finally, after centrifugation again at 13,000 r/min, the sediment was resuspended in glycine buffer (20 μL, pH 6.5). The acquired LM probes were stored at 4°C until further use.

### 2.4 Assemble of immunoassays

Five components make up an immunochromatography test strip: the PVC backing card (30 cm*6 cm), sample pad, conjugate pad, absorbent pad, and NC membrane (Ling et al., 2022). The PVC backing card was used as a supporting card, where the other pads were attached. The sample pad and conjugate pad (1 cm*0.2 cm) were pretreated with the solution buffer (PBS containing 5% BSA and 1% Tween-20). Goat anti-mouse IgG antibody and TTX-OVA (TTX-ovalbumin) were dispersed on the NC membrane to shape the control line (C line) and test line (T line), respectively, with a 5-mm interval, and then AuNF/LM probes were loaded onto the conjugate pad. Finally, the five components were assembled together, with an absorbent pad placed on the top and a sample pad at the bottom of the NC membrane. The complete immunochromatography test strip was chopped into 6-cm-long and 0.2-cm-wide strips for further use.

### 2.5 Evaluation of the developed immunoassays

μ-CTX KIIIA, αB-VxXXIVA-CTX, APETx_2, BDS-I, SN311, and OA (all the sample concentrations were 2000 ng/mL) were, respectively, added to the sample pads to assess the specificity of the prepared AuNF/LM-based strips. The sensitivity of AuNF/LM-based strips was tested by dropping TTX at different concentrations into the sample pads. The C and T lines of the test strip were recorded using immunochromato-reader. The standard curve was developed by drawing the B/B_0_ value against the logarithm of different concentrations of TTX, where B and B_0_ symbolize the ratio of T/C values with TTX and without TTX, respectively. The TTX concentration showing 90% B/B_0_ values according to the standard curve was measured to be the limit of detection (Li et al., 2020).

### 2.6 Stability of AuNF/LM probes and strips

The prepared AuNF probes and LM probes were stored at room temperature and 4°C, respectively, and the developed test strips were always stored at the room temperature. The test was performed every 3 days under the TTX and TTX-free conditions. Immunochromato-readers (Hamamatsu Photonics, Japan) were used to measure the absorbance of C and T lines to determine the stability of AuNF/LM probes and strips.

### 2.7 Application for real sample analysis

Yellow croaker, grass carp, perch, and cultured pufferfish (*Takifugu obscurus*) were randomly selected and pretreated as described previously with some modification (Shen et al., 2017). Briefly, muscle tissue (10 g) was extracted with 40 mL of 0.05% PBST (containing 0.3% Triton X-100, 0.01 M, pH 7.4). After allowed to stand for 10 min, the solution was centrifuged at 8,000 r/min for 10 min, and the supernatant was collected. The established immunochromatography test strips were used to analyze the TTX levels in different samples. In addition, an ELISA kit (Shanghai Yuanju Bio-Technology Center) was carried out to appraise the precision of the developed methods for TTX detection.

## 3 Results

### 3.1 Preparation of AuNF probes

**FIGURE 1:**
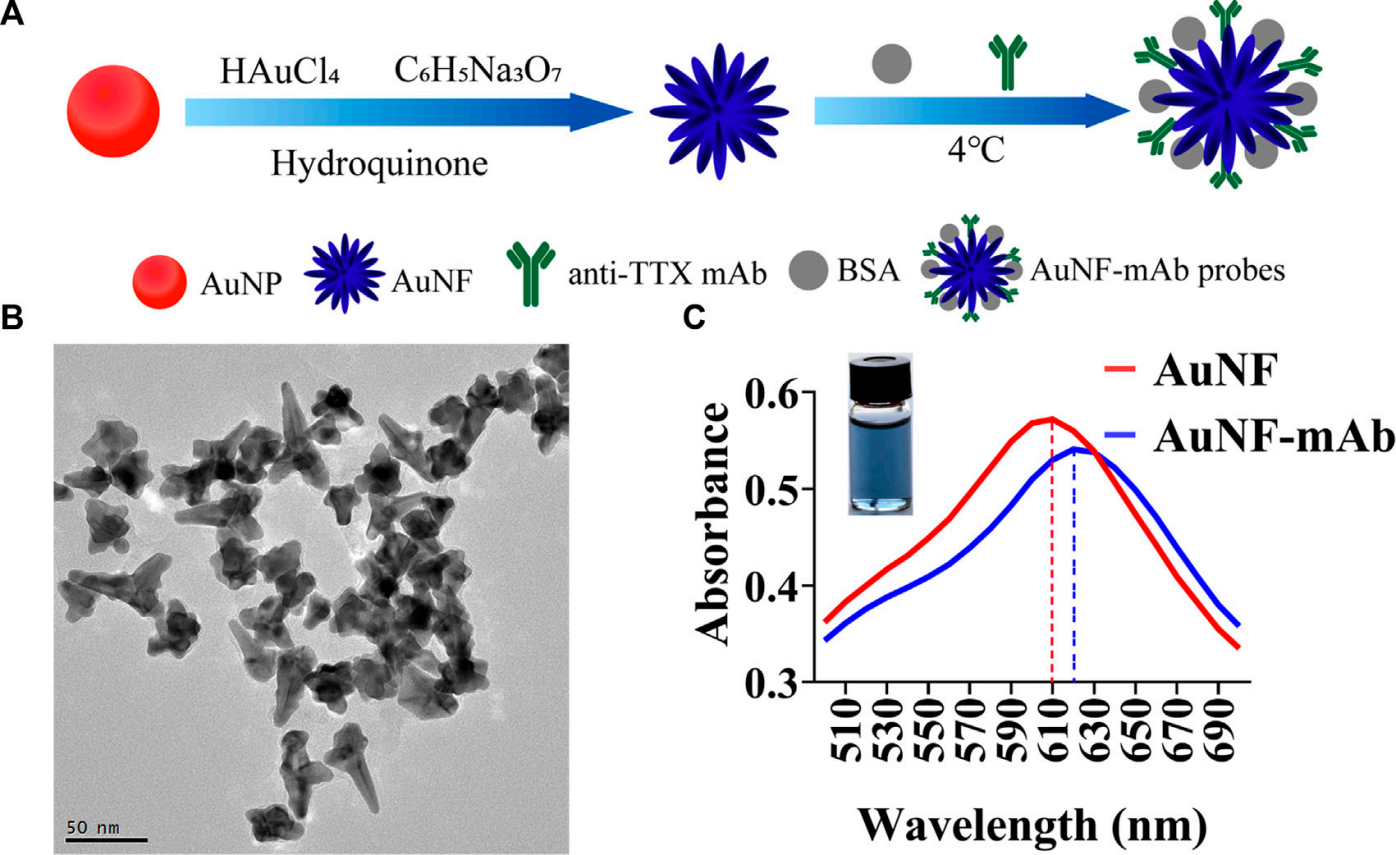
Synthesis and characterization of AuNF. **(A)** Synthesis process of AuNFs and AuNF probes. **(B)** Transmission electron microscopy (TEM) result of AuNFs. **(C)** Ultraviolet-visible absorption spectra of AuNFs (red) and AuNF probes (blue).

Traditional immunochromatographic test strips were often developed based on AuNP which was used as labeling material. Although, AuNP was easy to be synthesized and commonly used, the insufficient brightness of AuNP leads to low sensitivity of the test strips (Omidfar et al., 2013). For the purpose of improving the sensitivity of immunochromatographic test strips, multi-branched gold nanoflower (AuNF) exhibiting high brightness was prepared in the study. The schematic diagram of the preparation of AuNF and AuNF probes is shown in Figure 1A. AuNF was synthesized using AuNP as gold seeds (Zhang et al., 2019), and then anti-TTX mAb were labeled with the prepared AuNF to form AuNF probes. As shown in Figure 1B, nanoparticles with flower-like structures and multi-branches spreading out from a solid core were clearly observed. The size of AuNFs was determined to be approximately (60 ± 5) nm by counting the average diameter of 100 AuNFs. The features of the AuNF in TEM images were semblable to those described in a previous publication (Ji et al., 2015), meaning that AuNF with good quality was already prepared. Then, the preparation of the AuNF probe was carried out by labeling AuNF with anti-TTX mAb. It has been reported that the amount of anti-TTX mAb and pH of AuNF solution can affect AuNF probe synthesis (Wang et al., 2018). The optimum ratio of AuNF solution to anti-TTX mAb was determined to be 100: 1, and the optimum pH was adjusted using 2 μL of 0.1 M K_2_CO_3_ (Supplementary Figures S1A, B). The optimal concentrations of goat anti-mouse IgG and TTX-OVA were 0.67 mg/mL and 328 μg/mL, respectively (Supplementary Figures S2A, B). The optimal amount of AuNF probes was 4 μL (Supplementary Figure S2C). The maximum absorption peaks of AuNF and AuNF probes were observed at 610 nm and 620 nm, respectively (Figure 1C), indicating some shift between AuNF probes and AuNF. Moreover, dynamic light scattering (DLS) results also showed that there are some differences between the diameter of AuNF and that after the anti-TTX mAb labeling (Supplementary Figure S3A). All these results showed that AuNF and AuNF probes were successfully prepared and could be used for further study.

### 3.2 Representation of an AuNF-based strip

**FIGURE 2:**
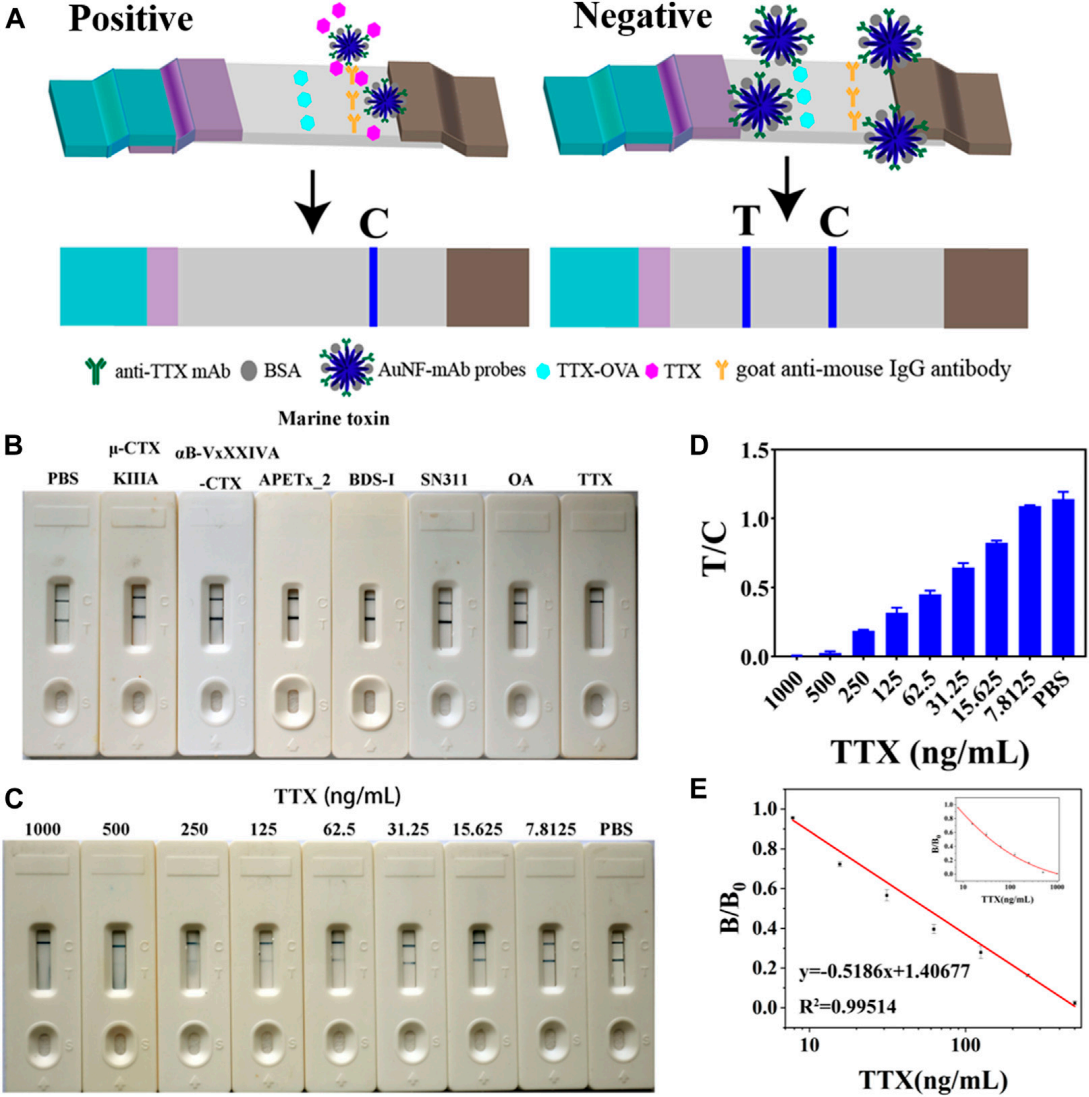
Representation of the AuNF-based strip (*n* = 3). **(A)** Schematic diagram of the developed AuNF-based strip. **(B)** Specificity of the AuNF-based strip by adding different marine toxins. **(C)** TTX with different concentrations effects on the color development of C and T lines on AuNF-based strips. **(D)** Immunochromato-reader absorbance values associated with the results of **(C)** for AuNF-based strips. **(E)** Linear range of TTX detection by AuNF- based strips.

The obtained AuNF probes were sprayed into the probe pads, and an AuNF-based strip was developed (Figure 2A). A process competition was conducted on AuNF-based strips. If samples contain TTX antigen, AuNF probes could first react with TTX, so there is no AuNF probe captured by coating antigen TTX-OVA immobilized on the NC membrane. Thus, only the C line displayed blue color. When there is no or little TTX contained in the samples, AuNF probes labeled with anti-TTX mAb could be specifically captured by TTX-OVA complete antigen, so both T and C lines showed blue color. To appraise the specificity of the AuNF-based strip test, other marine toxins (μ-CTX KIIIA, αB-VxXXIVA-CTX, APETx_2, BDS-I, SN311, and OA) were used in the experiment. An immunochromato-reader was used to read the absorbance of the T and C lines. As interpreted in Figure 2B, the developed AuNF-based test strip was used to detect TTX, and the result indicated no blue color on the T line when TTX was added to the sample pad. By decreasing the concentration of TTX, the blue color of the T line in Figures 2C, D gets darker and darker. According to Figure 2E, the concentration of TTX was linearly related in the range of 9.49–330.98 ng/mL and the LOD was confirmed to be 9.49 ng/mL. The calibration equation was Y = 0.5186X + 1.40677 (*R* ^2^ = 0.99514), where X represents the logarithm of the TTX concentration. The accuracy of the AuNF-based strip is shown in Supplementary Table S1. The recovery range rates of inter- and intra-assays were 91.96%–101.21% and 88.85%–102.88%, and the average recovery rate was 97.76% and 97.09%, respectively. The average coefficient of variation (CV) of inter- and intra-assays was less than 10%. It was reported that the LOD of the immunoassay method based on gold nanoparticles for TTX-spiked samples was measured to be 40 ng/mL and 10 μg/kg (Zhou et al., 2010; Li et al., 2020). Compared to the test strip using conventional AuNPs as signal reporters, the multi-branched AuNF of size (60 ± 5) nm could be used in LFICS, combining quantum dot nanobeads and AuNFs to significantly enhance the sensitivity of the test strip with a LOD of 0.2 ng/mL. However, the testing results could not be observed with the naked eye(Shen et al., 2017). The aforementioned results proved that the immunochromatographic test strip developed based on AuNF has the merits of high sensitivity and is easy to be observed with naked eyes.

### 3.3 Preparation of LM probes

**FIGURE 3:**
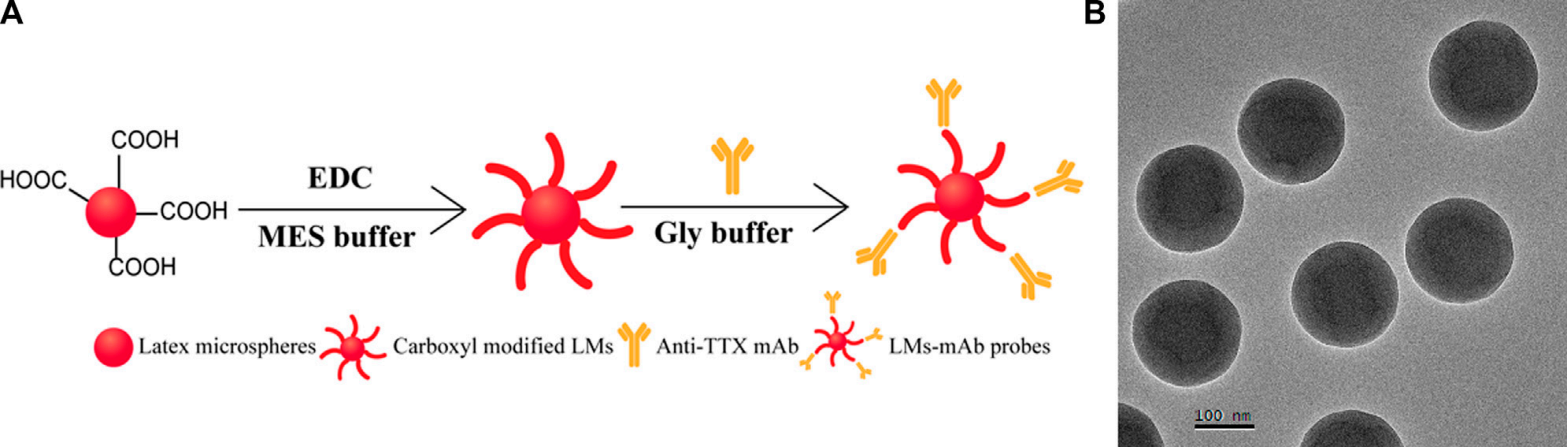
Synthesis and characterization of LM. **(A)** Synthesis process of LM probes. **(B)** TEM result of LMs.

LM not only has a large specific surface but also provides abundant carboxyl groups to bind with anti-TTX mAb (Li et al., 2021b), so LM was used as a sensitive label to conjugate with anti-TTX mAb for the development of an LM-based strip in this study. The schematic diagram of the preparation of LM and the LM probe is shown in Figure 3A. Red LM was carboxyl modified by EDC, and the activated LM was labeled with anti-TTX mAb. As shown in Figure 3B, the TEM images clearly showed round and uniform LMs. The size distribution of LMs was determined to be approximately (200 ± 5) nm. In addition, DLS results also showed that there are some differences between the diameter of LM and that after the anti-TTX mAb labeling (Supplementary Figure S3B). For the best performance of the LM probe, some factors, including the color of the latex microspheres, optimal pH values, and the amount of EDC and anti-TTX mAb, were optimized. The suitable color of latex microspheres for the TTX antibody is red (Supplementary Figure S4A). The optimal pH values for MES activation buffer and Gly buffer were 6 and 6.5, respectively (Supplementary Figures S4B, C). The quantities of EDC and anti-TTX mAb were optimized to be 1.5 mg and 10 μL, respectively (Supplementary Figures S4D, E). The concentrations of goat anti-mouse IgG (0.5 mg/mL) and TTX-OVA (32.80 μg/mL) (Supplementary Figures S5A, B), together with the amount of LM probes (4 μL), were also optimized (Supplementary Figure S5C).

### 3.4 Evaluation of an LM-based strip

**FIGURE 4:**
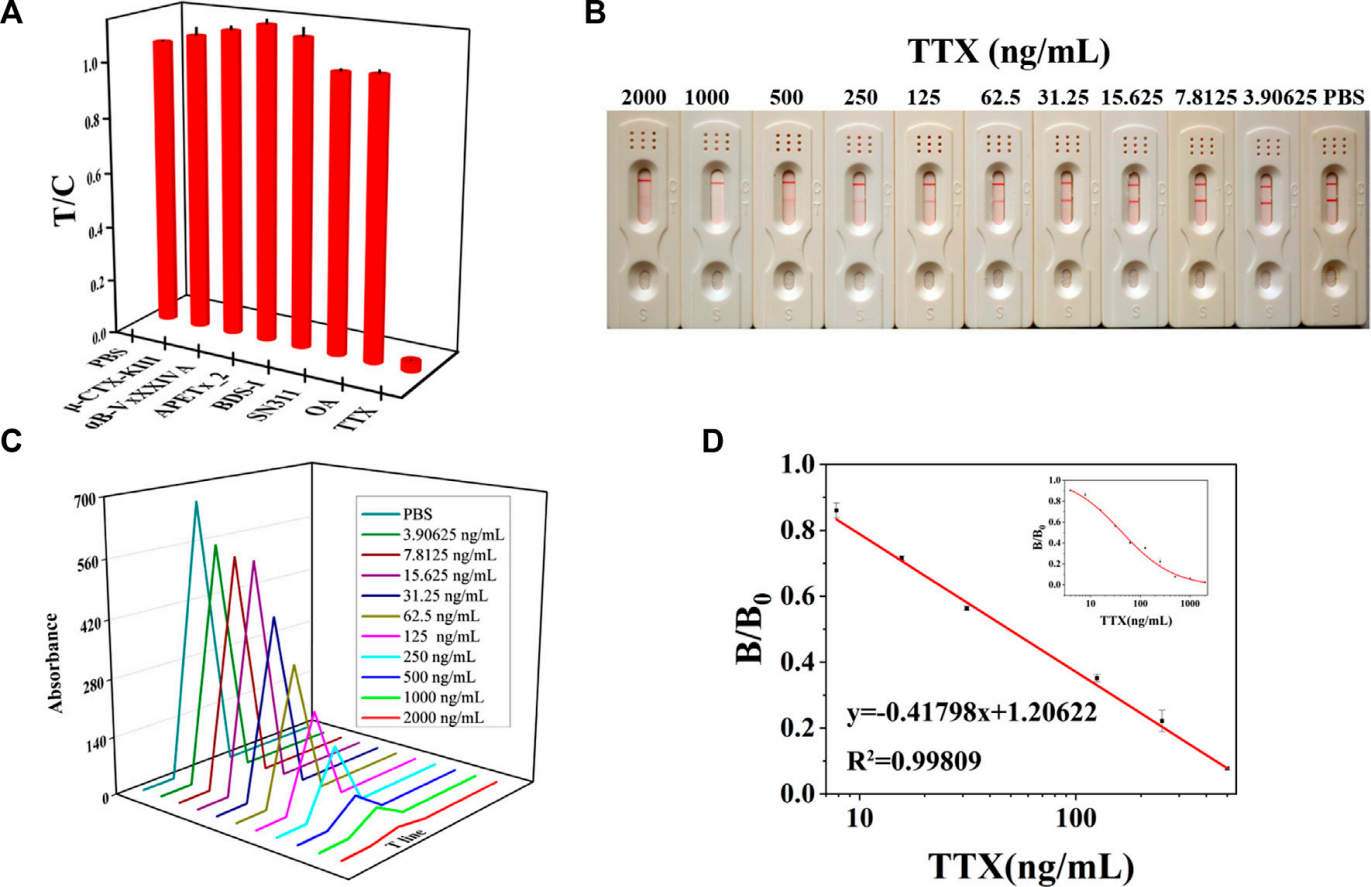
Representation of the LM-based strip (*n* = 3). **(A)** Specificity of the LM-based strip. **(B)** TTX with different concentrations was added to LM-based strips to test the sensitivity results. **(C)** T value of LM-based strips using the immunochromato-reader corresponding to the results of **(B)**. **(D)** Linear range of TTX detection by LM-based strips.

Then, an LM-based strip test was developed based on the LM probe, which was prepared under optimal conditions. The principle of the LM-based strip test was similar to that of the AuNF-based strip. The specificity results showed that the prepared LM-based strip had great specificity, for it did not cross-react with any other marine toxins except for TTX (Figure 4A). With increasing TTX concentrations, the red color shown in Figure 4B becomes lighter and lighter according to the LM-based strip test. As illustrated in Figures 4C, D, the calibration equation was Y = 0.41798X + 1.20622 (*R* ^2^ = 0.99809) with a linear range of 5.40–443.19 ng/mL and LOD of 5.40 ng/mL. The precision of the LM-based strip is illustrated in Supplementary Table S2. The recovery rates of inter- and intra-assays were in the range of 95%–105.09% and 83.74%–107.90%, and the average recovery rate was 99.48% and 96.08%, respectively. The average CV of inter- and intra-assays was less than 10%. Compared to the sensitivity (LOD = 9.49 ng/mL or 9 μg/kg) of AuNF-based strips developed previously, LM as a signal reporter showed more sensitivity (LOD = 5.40 ng/mL or 5 μg/kg). The possible reason for the higher sensitivity of LM-based strips may be due to the larger size of LMs of (200 ± 5) nm than that of AuNF of (60 ± 5) nm. In addition, the LOD of both AuNF- and LM-based strips were lower than the maximum permissible limit for TTX (2.2 mg/kg) set by Japan and TTX (44 μg/kg) set by the Netherlands (Lago et al., 2015; Kosker et al., 2018). Our results indicated that the developed LM-based strip test with the advantages of high specificity, low detection limit (lower than the national standards of Japan and the Netherlands), wide detection range (5.40–443.19 ng/mL), and rapid detection (10 min) could meet the detection requirements of tetrodotoxin.

### 3.5 Stability of AuNF- and LM-based strips

**FIGURE 5:**
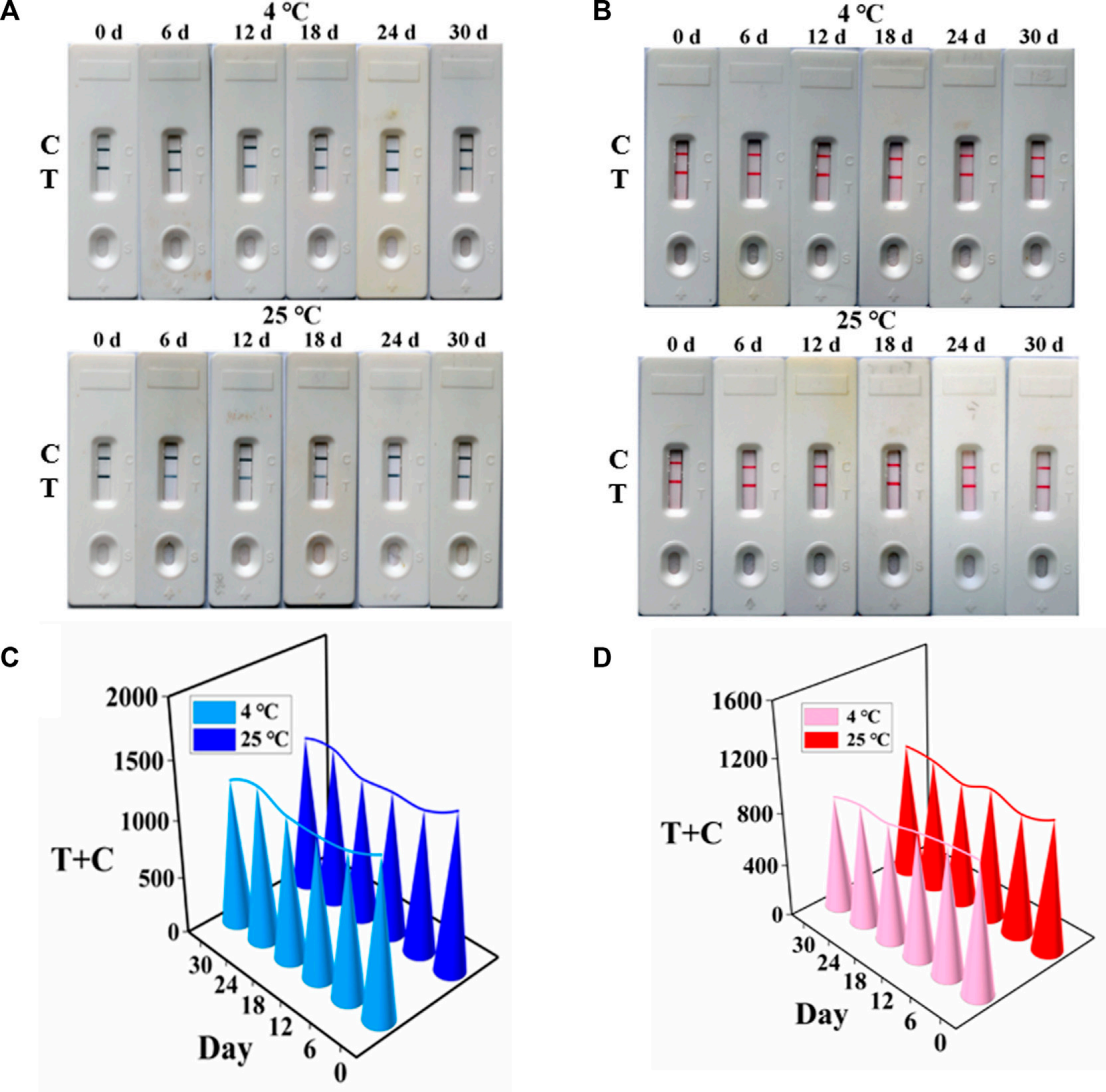
Stability of AuNF/LM-based strips (*n* = 3). **(A)** Stability results of AuNF-based strips. **(B)** Stability results of LM-based strips. **(C)** T + C values of the developed AuNF-based strip. **(D)** T + C values of the LM-based strip.

To appraise the potential of the developed AuNF/LM-based strip for the determination of TTX, the stability of AuNF and LM probes at 4°C and room temperature were measured, respectively. As shown in Figures 5A, B for the TTX-free group, AuNF probes and LM probes were very stable when stored at 4°C and 25°C, and the bands were clearly observed within 1 month. The values (T + C) of the developed strip test were stable (Figures 5C, D). For the TTX group, AuNF and LM probes were also stored at 4°C and 25°C, respectively, and the results in Supplementary Figures S6A, B showed that the developed immunochromatography test strips based on AuNF- and LM-enhanced signal probes have good stability and provide great potential for commercial application.

### 3.6 Application in real sample analysis

**FIGURE 6:**
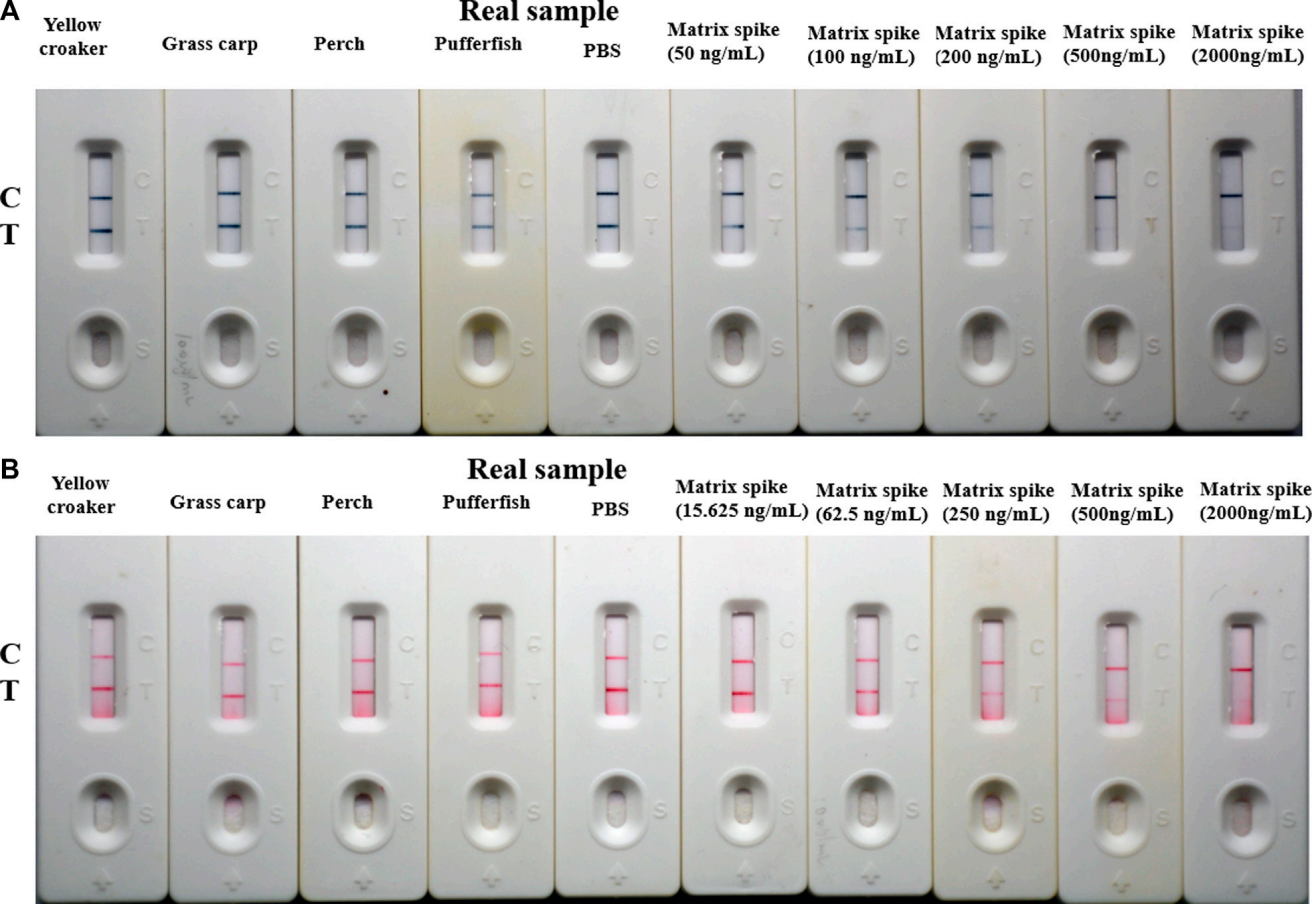
Real sample testing of the established AuNF/LM-based strips (*n* = 3). **(A)** Detection results of real samples determined by AuNF-based strips. **(B)** Testing results of real samples determined by LM-based strips.

The matrix effect was evaluated by spiking various concentrations of TTX in the range of 0–1,000 ng/mL into PBS and TTX-free sample extracts. As shown in Supplementary Figure S7, there exists little matrix effect. For the best application of the prepared test strip to detect TTX, we measured four actual samples (including yellow croaker, grass carp, perch, and pufferfish) and the matrix spiked with 50, 100, 200, 500, and 2000 ng/mL TTX for AuNF-based strips and 15.625, 62.5, 250, 500, and 2000 ng/mL TTX for LM-based strips. As with PBS (negative control), when yellow croaker, grass carp, perch, or pufferfish extracts were added to the AuNF/LM-based strips, two blue/red lines appeared on the NC membrane. The T line becomes lighter and lighter as the spiked TTX concentration increases (Figures 6A, B), indicating that the AuNF/LM-based strips have good reliability and practicability for TTX detection. Moreover, the reliability of the established methods was further confirmed by commercially available ELISA kit, and the TTX detection results of the actual samples and the matrix spiked with TTX (Supplementary Table S3) indicated that the results of AuNF/LM-based strip methods were in agreement with those of the ELISA assay.

## 4 Conclusion

In this study, immunochromatographic test strips based on enhanced signal reporters including AuNF and LM were successfully established to determine TTX. The developed immunochromatographic test strips have good specificity and sensitivity for the detection of TTX (with a LOD of 9.49 ng/mL for the AuNF-based strip and 5.40 ng/mL for the LM-based strip). The detection results could be obtained within 10 min by visualization. In short, we have successfully established two immunochromatographic test strips with the merits of high sensitivity and high speed for the determination of TTX, which has good potential for application in food safety.

## Data Availability

The original contributions presented in the study are included in the article/Supplementary Material; further inquiries can be directed to the corresponding authors.
