# Supplementary material for: Sensitive and rapid detection of tetrodotoxin based on gold nanoflower-and latex microsphere-labeled monoclonal antibodies
Source: Front Bioeng Biotechnol. 2023 May 16;11:1196043. doi: 10.3389/fbioe.2023.1196043 (PMC10227513; doi:10.3389/fbioe.2023.1196043)
Supplement: Supplementary file 1 [file DataSheet1.docx]

Supporting Information

Sensitive and rapid detection of tetrodotoxin based on gold nanoflower-and latex microsphere-labeled monoclonal antibodies

Yongming Huang, Aidi Xu, Yang Xu, Huijuan Wu, Menghan Sun, Lakshani Madushika, Rongzhi Wang, Jun Yuan, Shihua Wang^*^, Sumei Ling^*^

The Ministry of Education Key Laboratory of Biopesticide and Chemical Biology, Fujian Key Laboratory of Pathogenic Fungi and Mycotoxins, and School of Life Sciences, Fujian Agriculture and Forestry University, Fuzhou 350002, China

*Corresponding author. Tel and Fax: +086 (591) 87984471.

E-mail: wshyyl@sina.com (S.H. Wang) or Lsmpu2008@163.com (S.M. Ling)


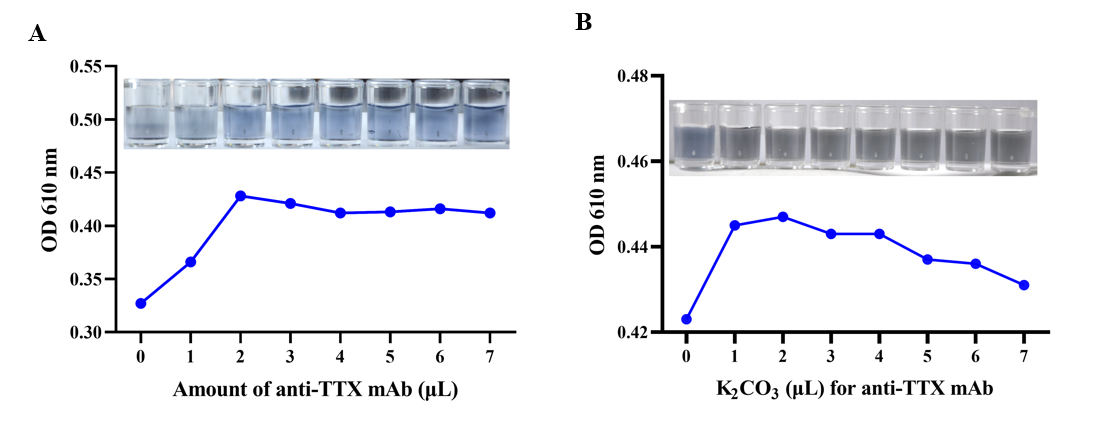


**Fig. S1** Effect of the amount of anti-TTX mAb and pH value on AuNF probes. (A) Effect of the amount of anti-TTX mAb on AuNF probes. (B) Effect of the pH value on AuNF probes.


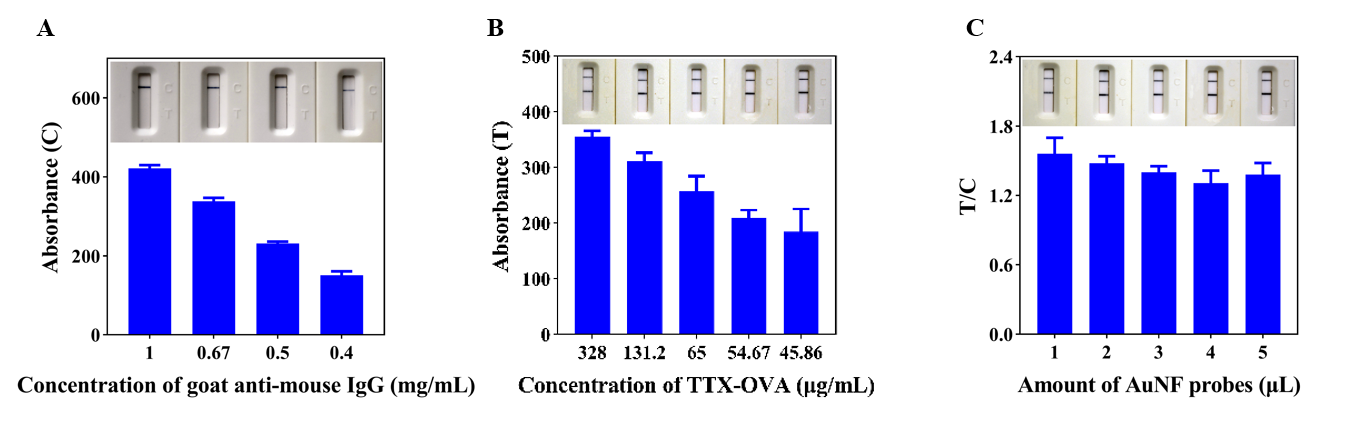


**Fig. S2** Effect of goat anti-mouse IgG, TTX-OVA and AuNF probes on the performance of AuNF-based strip. (A) Effect of the concentration of goat anti-mouse IgG on the C value. (B) Effect of the concentration of TTX-OVA on the T value. (C) Effect of the amount of AuNF probes on T/C value.


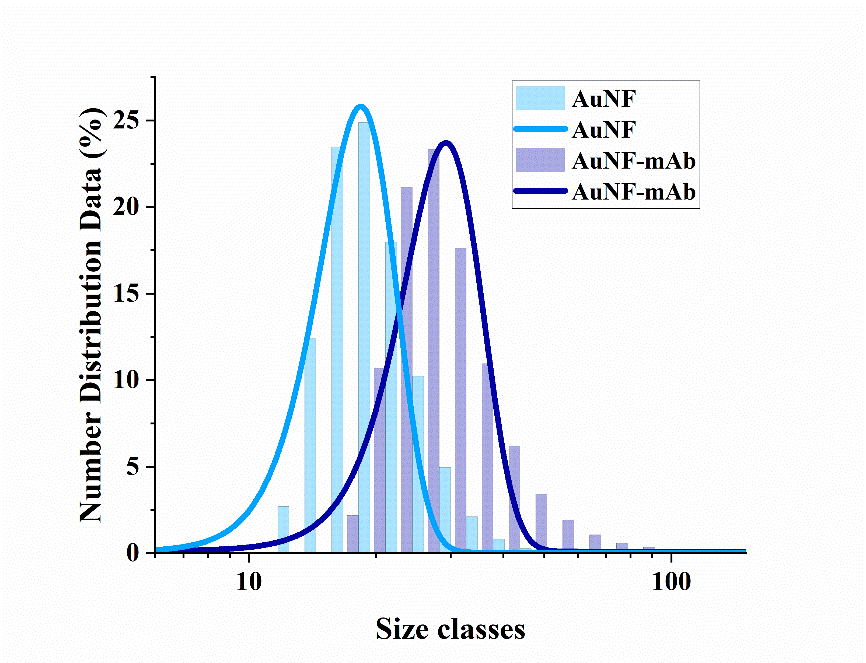

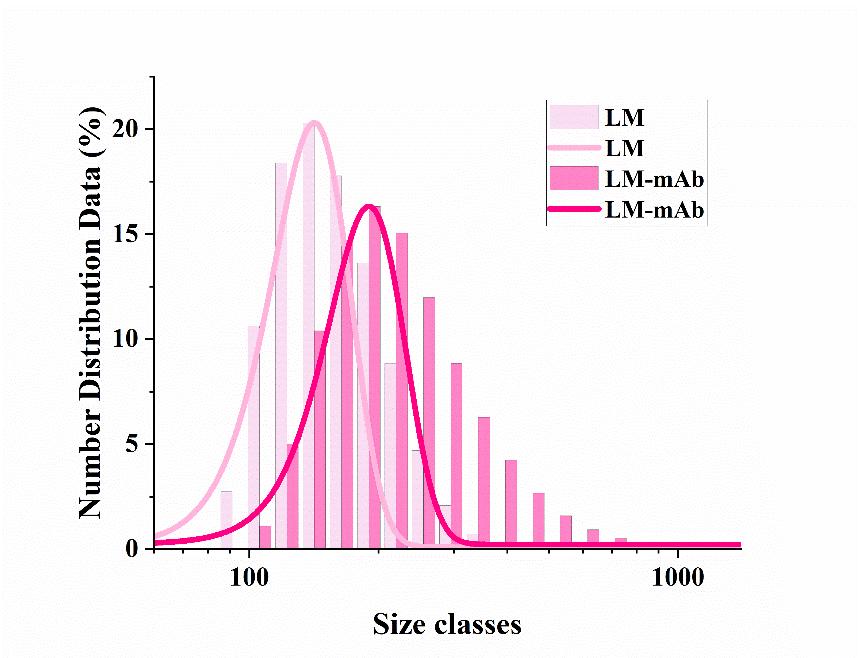


**A**

**B**

**Fig. S3** Dynamic light scattering (DLS) results. (A) DLS results of AuNF and AuNF-mAb. (B) DLS results of LM and LM-mAb.


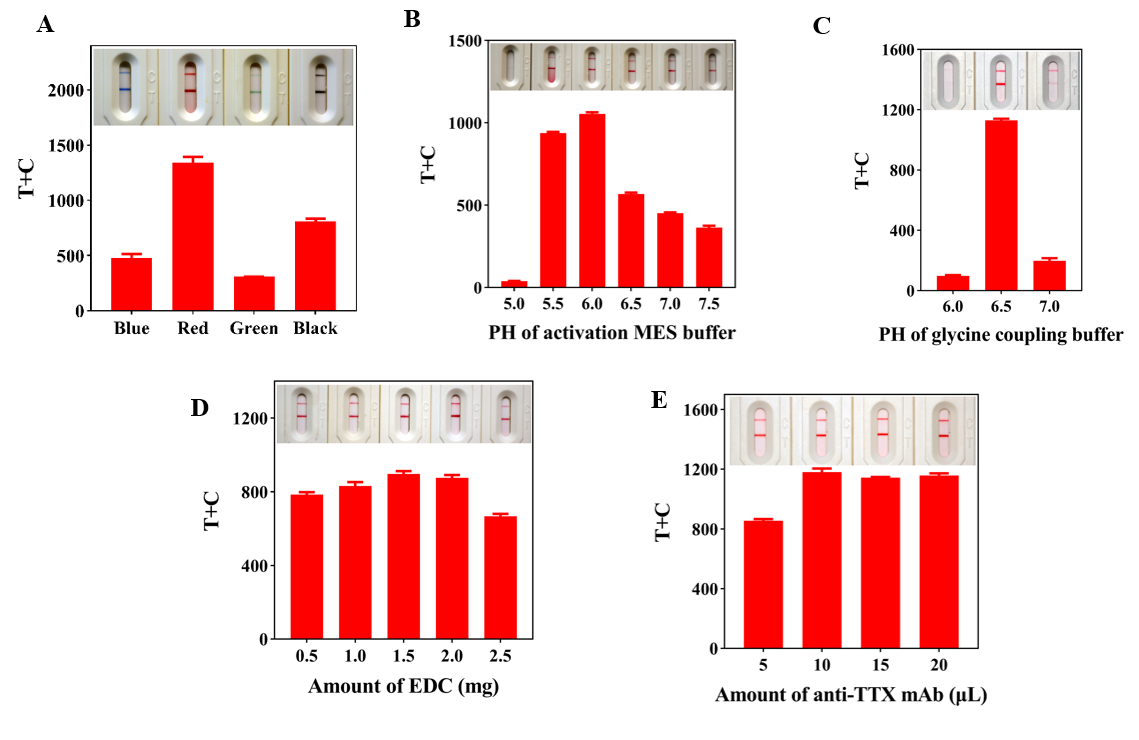


**Fig. S4** Effect of color, the pH value of MES buffer and Gly buffer, and the amount of EDC and anti-TTX mAb on the performance of LM-based strip. (A) Effect of the color on the T+C value. (B) Effect of the pH value of MES buffer on the T+C value. (C) Effect of the pH value of Gly coupling buffer on the T+C value. (D) Effect of the amount of EDC on performance of LM-based strip. (E) Effect of the amount of anti-TTX mAb on performance of LM-based strip.


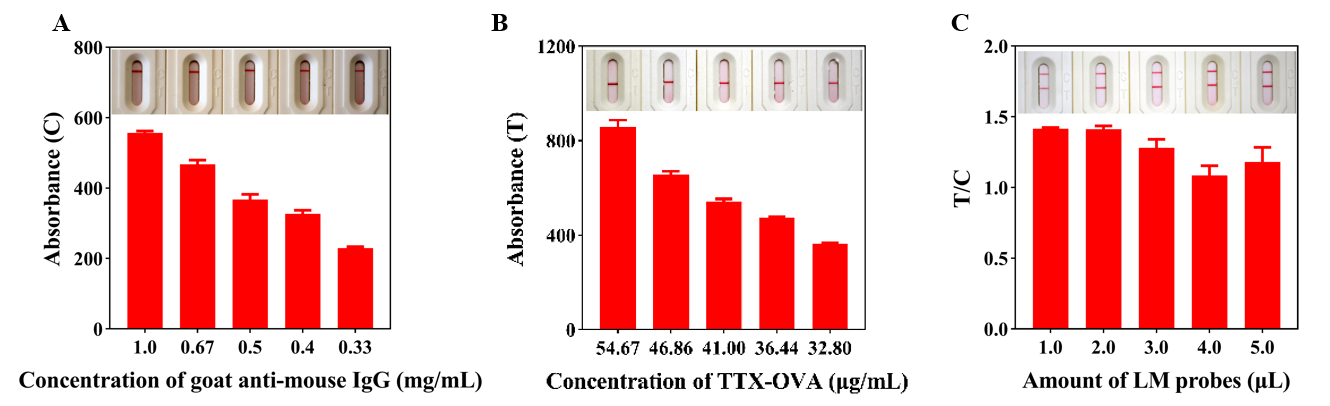


**Fig. S5** Effect of goat anti-mouse IgG, TTX-OVA and LM probes on the performance of LM-based strip. (A) Effect of the concentration of goat anti-mouse IgG on the C value. (B) Effect of the concentration of TTX-OVA on the T value. (C) Effect of the amount of LM probes on T/C value.


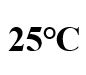

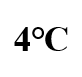

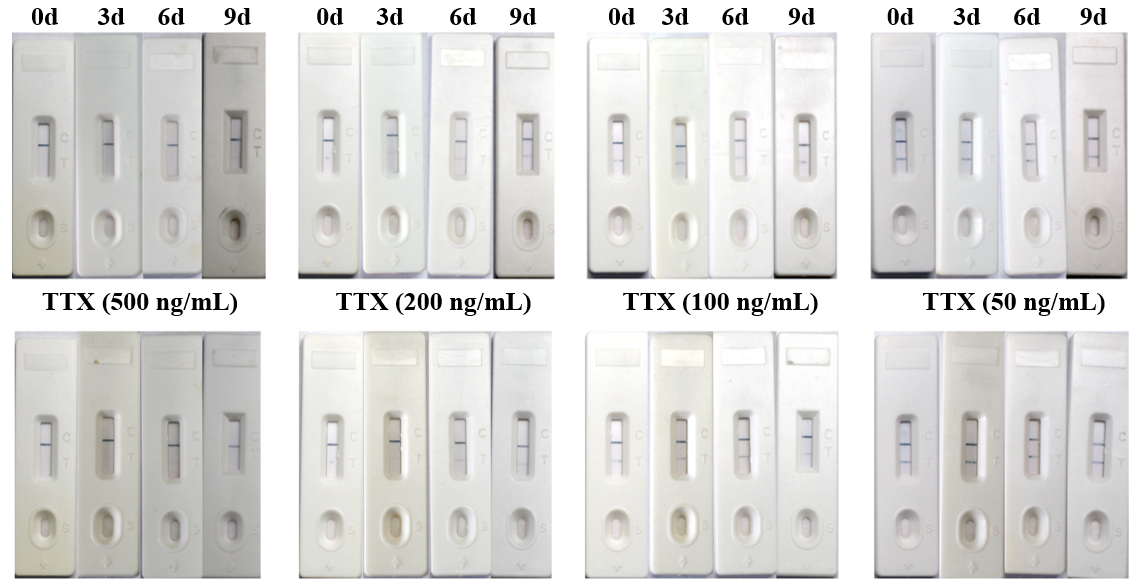


**B**

**A**


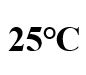

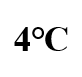

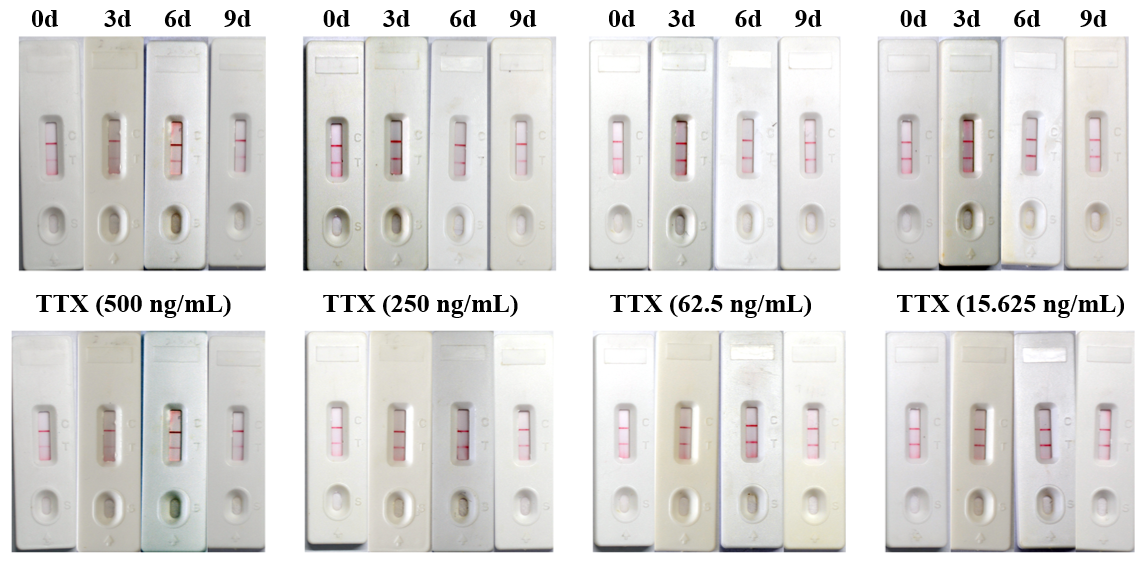


**Fig. S6** Stability of AuNF/LM-based strips. (A) The stability results of AuNF-based strips in TTX group. (B) The stability results of LM-based strips in TTX group.


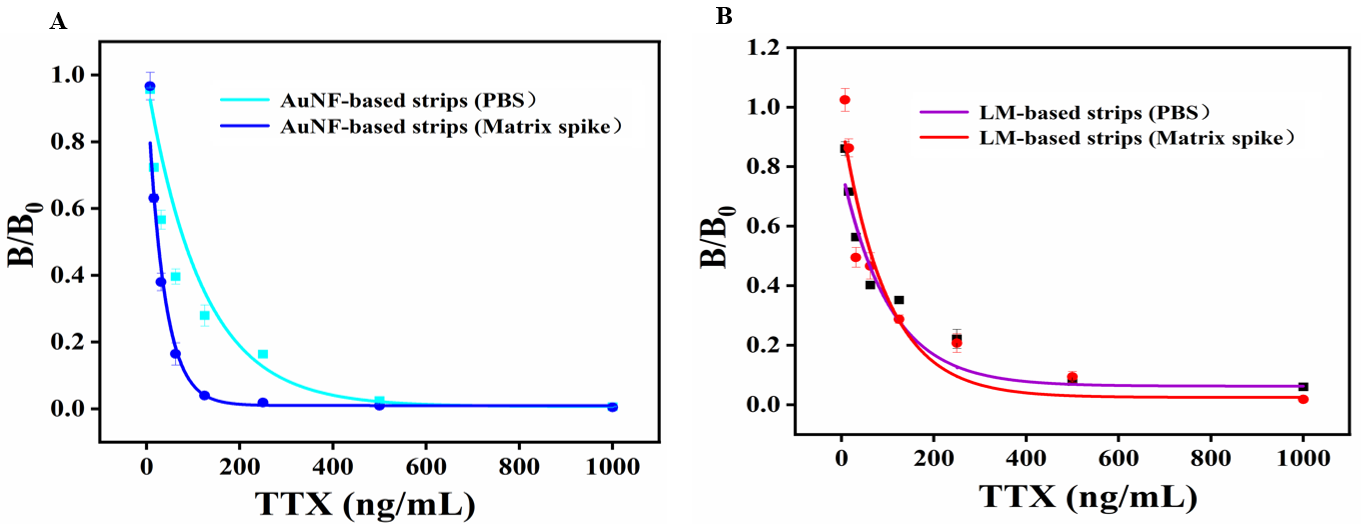


**Fig. S7** Evaluation of matrix effect of the developed methods. (A) Matrix effect of AuNF-based strips. (B) Matrix effect of LM-based strips.

**Tab. S1** Accuracy of the AuNF-based strips

| Spiked | | Intra-assay | | | |  | Inter-assay | | | |
| --- | --- | --- | --- | --- | --- | --- | --- | --- | --- | --- |
| Level  (ng/mL) | | n | Measured  (ng/mL) | Recovery  (%) | CV  (%) |  | n | Measured  (ng/mL) | Recovery  (%) | CV  (%) |
| 200 | | 3 | 199.07±3.23 | 99.53±1.62 | 1.62 |  | 3 | 202.42±2.39 | 101.21±2.39 | 1.18 |
| 100 | | 3 | 102.88±4.41 | 102.88±4.41 | 4.29 |  | 3 | 100.12±1.97 | 100.12±1.97 | 1.97 |
| 50 | | 3 | 44.42±1.13 | 88.85±2.25 | 2.54 |  | 3 | 45.98±2.52 | 91.96±2.52 | 5.50 |
| Average | |  | - | 97.09±2.76 | 2.82 |  |  | - | 97.76±2.29 | 2.88 |
|  | Recovery= (Detection concentration/Spiked concentration)×100%.  CV= (Standard Deviation/mean)×100%. | | | | | | | | | |

**Tab. S2** Accuracy of the LM-based strips

| Spiked | | Intra-assay | | | |  | Inter-assay | | | |
| --- | --- | --- | --- | --- | --- | --- | --- | --- | --- | --- |
| Level  (ng/mL) | | n | Measured  (ng/mL) | Recovery  (%) | CV  (%) |  | n | Measured  (ng/mL) | Recovery  (%) | CV  (%) |
| 250 | | 3 | 209.36±3.44 | 83.74±1.38 | 1.64 |  | 3 | 237.5±19.92 | 95±7.97 | 8.39 |
| 62.5 | | 3 | 60.37±5.72 | 96.60±9.14 | 9.47 |  | 3 | 61.49±1.26 | 98.39±2.01 | 2.05 |
| 15.625 | | 3 | 16.86±1.50 | 107.90±9.60 | 8.92 |  | 3 | 16.42±0.64 | 105.09±4.09 | 3.92 |
| Average | |  | - | 96.08±6.71 | 6.68 |  |  | - | 99.48±4.67 | 4.79 |
|  | Recovery= (Detection concentration/Spiked concentration)×100%.  CV= (Standard Deviation/mean)×100%. | | | | | | | | | |

**Tab. S3** Determination of TTX concentration in samples by ELISA, AuNF-based strips and LM-based strips

| Sample | ELISA kit (ng/mL) | AuNF-based strips | LM-based strips |
| --- | --- | --- | --- |
| Yellow croaker | ND | - | - |
| Grass carp | ND | - | - |
| Perch | ND | - | - |
| Pufferfish | ND | - | - |
| Matrix spike | 410.88 | 490.44 | 485.23 |
| PBS | ND | - | - |

ND: Non-detected

-: Negative
